# HyperTRCSS: A hyperspectral time-resolved compressive sensing spectrometer for depth-sensitive monitoring of cytochrome-c-oxidase and blood oxygenation

**DOI:** 10.1117/1.JBO.29.1.015002

**Published:** 2024-01-24

**Authors:** Natalie C. Li, Seva Ioussoufovitch, Mamadou Diop

**Affiliations:** a Western University, School of Biomedical Engineering, Faculty of Engineering, London, Ontario, Canada; b Western University, Schulich School of Medicine and Dentistry, Department of Medical Biophysics, London, Ontario, Canada; c Lawson Health Research Institute, Imaging Program, London, Ontario, Canada

**Keywords:** near-infrared spectroscopy, hyperspectral, time-resolved, cytochrome-c-oxidase, blood-yeast phantom

## Abstract

**Significance:**

Hyperspectral time-resolved (TR) near-infrared spectroscopy offers the potential to monitor cytochrome-c-oxidase (oxCCO) and blood oxygenation in the adult brain with minimal scalp/skull contamination. We introduce a hyperspectral TR spectrometer that uses compressive sensing to minimize acquisition time without compromising spectral range or resolution and demonstrate oxCCO and blood oxygenation monitoring in deep tissue.

**Aim:**

Develop a hyperspectral TR compressive sensing spectrometer and use it to monitor oxCCO and blood oxygenation in deep tissue.

**Approach:**

Homogeneous tissue-mimicking phantom experiments were conducted to confirm the spectrometer’s sensitivity to oxCCO and blood oxygenation. Two-layer phantoms were used to evaluate the spectrometer’s sensitivity to oxCCO and blood oxygenation in the bottom layer through a 10 mm thick static top layer.

**Results:**

The spectrometer was sensitive to oxCCO and blood oxygenation changes in the bottom layer of the two-layer phantoms, as confirmed by concomitant measurements acquired directly from the bottom layer. Measures of oxCCO and blood oxygenation by the spectrometer were highly correlated with “gold standard” measures in the homogeneous and two-layer phantom experiments.

**Conclusions:**

The results show that the hyperspectral TR compressive sensing spectrometer is sensitive to changes in oxCCO and blood oxygenation in deep tissue through a thick static top layer.

## 1 Introduction

Near-infrared spectroscopy (NIRS) is now widely used for neuromonitoring owing to its portability, safety, and sensitivity to changes in cerebral blood oxygenation and metabolism.^1^^–^^7^ The technique is based on measuring the effects of light–tissue interaction, which can be used to quantify tissue optical properties, such as its absorption and scattering coefficients.^8^ In the NIR range (650 to 1100 nm), the tissue light absorbers (i.e., chromophores) of clinical importance are water, hemoglobin, lipid, and cytochrome-c-oxidase (CCO)—an enzyme involved in cellular oxidative metabolism.^9^ NIRS is highly sensitive to the oxygenation state of hemoglobin and can easily quantify changes in the concentrations of oxy- and deoxy-hemoglobin (HbO and Hb) using their distinct optical signatures.^10^ Although NIRS measures of blood oxygenation are commonly used as a surrogate for tissue oxygen metabolism,^11^ the redox state of CCO is believed to be a superior biomarker as it reflects tissue oxygen metabolism at the cellular level.^12^ Notably, CCO is the terminal electron acceptor in the mitochondrial electron transport chain, and its redox state is dependent on oxygen availability and electron supply.^13^ Further, hemoglobin deoxygenates readily with small reductions in oxygen tension, whereas CCO is relatively insensitive to the fluctuations that occur under normal physiological conditions.^13^^–^^15^ CCO reduces only under extreme hypoxic conditions;^13^^–^^15^ thus, it may be a better indicator of impending brain injury than blood oxygen saturation.^12^

Since the total concentration of CCO is typically constant over a multi-hour monitoring period, resolving changes in only one of its redox states is sufficient to monitor changes in metabolism.^16^ The difference in the absorption spectra of the oxidized and reduced forms of CCO (ox-redCCO) can be used to quantify changes in the concentration of oxidized CCO (oxCCO).^16^ However, the broad spectral shape of the ox-redCCO spectrum and the low concentration of CCO relative to hemoglobin present challenges for NIRS monitoring. The ox-redCCO difference spectrum is broad, peaking at 830 nm and spanning tens of wavelengths;^17^^–^^19^ thus, instruments must be sensitive over a wide spectral range to fully characterize its shape. Further, the concentration of CCO in the brain is 10 to 20 times smaller than the concentration of hemoglobin.^13^^,^^16^ The oxCCO signal is therefore highly sensitive to cross talk with hemoglobin, and changes in light attenuation due to CCO are easily masked by the larger changes caused by hemoglobin.^20^ Cross talk can be reduced by increasing the number of wavelengths used for monitoring oxCCO.^20^^–^^22^ Thus, the current “gold standard” for monitoring oxCCO is to use a broadband continuous-wave (CW) system that measures light attenuation across the 780 to 900 nm spectral range in 1-nm increments; this is known as a “hyperspectral” approach.^16^^,^^20^^–^^23^

Several studies have demonstrated that oxCCO can be monitored in piglets and neonates using CW systems;^5^^,^^7^^,^^24^ however, extending the technique to adults has been hampered by contamination of brain-related signals by the thick adult extracerebral layers (ECL; scalp and skull).^6^^,^^25^^,^^26^ In adults, substantial light absorption and scattering occur in these surface layers, limiting light penetration into deeper tissues and reducing sensitivity to the brain.^27^^–^^29^ Multi-distance measurements have been proposed to account for the ECL contribution to the NIRS signal,^25^^,^^26^^,^^30^^,^^31^ wherein the signal from a short source–detector distance is regressed from a long source–detector distance measurement. However, this approach assumes that the ECL contribution to both signals is similar, which may not be the case due to local variations in scalp hemodynamics.^32^ An alternative approach to improve sensitivity to the brain is time-resolved (TR) NIRS. TR-NIRS sends light pulses into the head and precisely measures the times-of-flight (TOFs) of the detected photons. TR acquisitions provide a measure of optical pathlength,^33^^,^^34^ enable quantification of both tissue absorption and scattering properties,^35^^,^^36^ and the signal contributions of the ECL can be separated from those of the brain by discriminating early- from late-arriving photons.^37^^–^^41^ The improved sensitivity of TR-NIRS to the adult brain is well-known, but most TR-NIRS devices only provide measurements at 2 to 3 wavelengths, which is sub-optimal for oxCCO monitoring.

Although several hyperspectral TR-NIRS systems have been developed, they typically suffer from trade-offs between spectral resolution, spectral range, and acquisition time. Modern systems use pulsed supercontinuum lasers for illumination^42^ and time-correlated single-photon counting (TCSPC) modules for detection.^43^^–^^50^ While streak cameras have historically been used for detection,^51^^–^^54^ modern systems have adopted TCSPC-enabled detection modules for their superior sensitivity and dynamic range.^42^ These TCSPC-based systems can generally be grouped into two categories based on their acquisition scheme: parallel detection or scanning. In parallel detection, the sample is illuminated with all wavelengths at once, and the detected light is dispersed onto a detector array, typically with 16 channels. Parallel detection offers faster acquisition times but suffers from the trade-off between spectral range and resolution; the wider the range, the more wavelengths per channel, and the worse the resolution.^44^^,^^48^^,^^50^ In sequential scanning schemes, a monochromator is used during either the illumination or detection stage to select the wavelengths for acquisition, one at a time. Sequential scanning offers high spectral resolution but suffers from longer acquisition times when scanning over a wide spectral range (often >1 min to scan 100 wavelengths).^43^^,^^45^^,^^47^

To address these trade-offs, we previously proposed compressive sensing (CS) as a fast method of acquiring TR-NIRS data without compromising on spectral range or resolution.^55^ By applying high compression rates (up to 90%), the acquisition time per spectrum is reduced from several minutes to seconds. Recently, we have developed a hyperspectral TR CS spectrometer that can acquire 170-point spectra over 675 to 875 nm in 20 s while exhibiting no differences from spectra acquired without compression.^56^ We hypothesize that this device is sensitive to dynamic changes in blood oxygenation and oxCCO in deep (>10  mm) tissue (e.g., adult brain) through a thick top layer (e.g., ECL). In this work, we compare the device’s performance against a commercial CW spectrometer in homogeneous blood-yeast phantoms and then test the hypothesis in two-layer blood-yeast tissue-mimicking phantoms. In addition, Monte-Carlo (MC) simulations of the homogeneous and two-layer phantom experiments were conducted to refine the TR and CW data analysis pipelines and provide a point of reference for the experimental results.

## 2 Methods

### 2.1 Instrumentation and Data Acquisition Scheme

The initial development of the hyperspectral TR CS spectrometer was reported in Ref. 55. The spectrometer was rebuilt with modifications to address key limitations of the initial system. First, to better capture the spectral features of Hb, HbO, and oxCCO, the coupling between the optical components was optimized to increase the spectral range from 710 to 830 nm to 675 to 875 nm. Second, the photomultiplier tube (PMT) detector was replaced with a hybrid PMT to avoid detector afterpulsing and improve the quality of late-photon data. Third, the entire system was built onto a cart for portability. Further, the data acquisition in Ref. 55 was not optimized for monitoring dynamic physiological changes; here we use a higher compression rate to enable faster acquisitions.

**Fig. 1:**
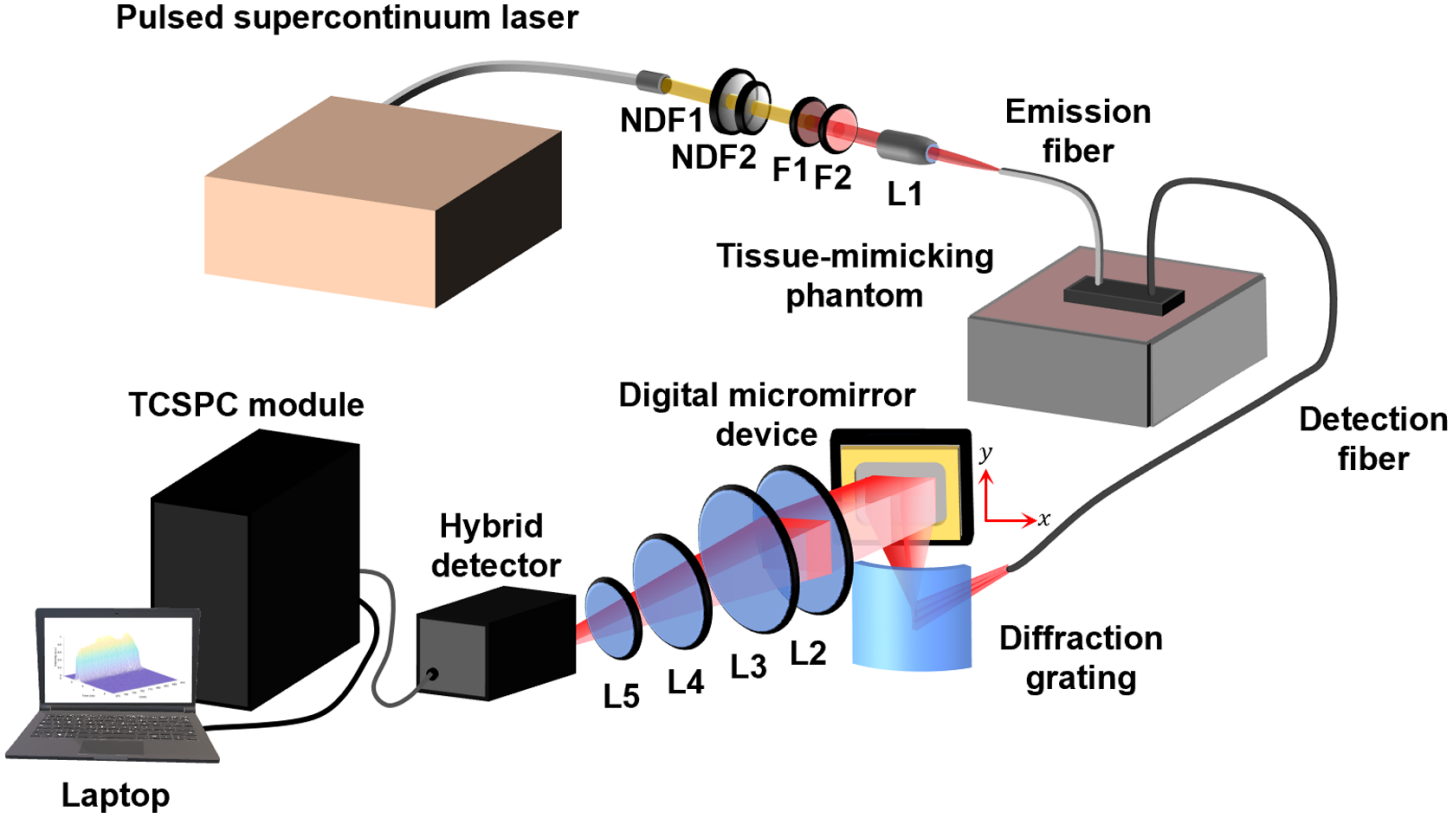
Schematic of the modified TR spectrometer (HyperTRCSS). The output from the pulsed supercontinuum laser (YSL or NKT source) is first attenuated by neutral density filters (NDF1, NDF2), then bandlimited by spectral filters (F1, F2). A microscope objective (L1) couples the filtered light to an emission fiber. The emission probe delivers the light to the sample—a tissue-mimicking phantom in this example—and a detection probe collects diffuse light reflected from the sample. The collected light is dispersed by a diffraction grating and directed by the digital micromirror device through a series of lenses (L2–L5) to a hybrid PMT detector connected to a TCSPC module. Reproduced from Ref. 56.

To show reproducibility, we tested the spectrometer with two different supercontinuum lasers: a YSL supercontinuum source SC-PRO 7 (400 to 2400 nm; YSL Photonics, China) and a more expensive SuperK FIANIUM FIR-20 (400 to 2500 nm; NKT Photonics, Denmark). The YSL and NKT lasers were operated at repetition rates of 80 and 78 MHz, respectively. Figure 1 shows a schematic of the modified TR spectrometer. The output of the light source is first transmitted through spectral filters (FELH0650, FES0950, Thorlabs Inc.) to limit the bandwidth to 650 to 950 nm before illumination of the sample. Variable and absorptive neutral density filters (NDC-50C-4M and NE40A, Thorlabs Inc.) are used to limit the photon count rate to 1% of the laser’s repetition rate. The filtered beam is then coupled by a microscope objective (RMS10X, Thorlabs Inc.) to a 3 mm diameter bundle of multimode fibers, which guides light to the sample. Diffusely reflected light from the sample is collected by a round-to-linear fiber bundle (D=1.55  mm [round], 0.25  mm×7.50  mm [linear], NA = 0.55, ScienceTech Inc., Canada) and directed to a concave holographic grating (f=100  mm, 520  lines/mm, 16  nm/mm dispersion, ScienceTech Inc., Canada). The grating spectrally disperses the light such that wavelength varies in the x direction. The grating output is imaged onto a digital micromirror device (DMD; DLi4130.7” VIS XGA High-Speed Development Kit, active area W×H=14.2  mm×10.7  mm, Digital Light Innovations), which spatially encodes the spectrum. The DMD output is focused by a series of lenses (FRP232, LC1715-B, LB1757-B, LB1761, Thorlabs Inc.) into a single channel hybrid PMT detector (PMA Hybrid 50, PicoQuant GmbH, Germany) coupled to a TCSPC module (SPC-130, Becker & Hickl GmbH, Germany). Hereafter, the modified spectrometer will be referred to as HyperTRCSS (hyperspectral time-resolved compressive sensing spectrometer; “Hyper-tricks”).

**Fig. 2:**
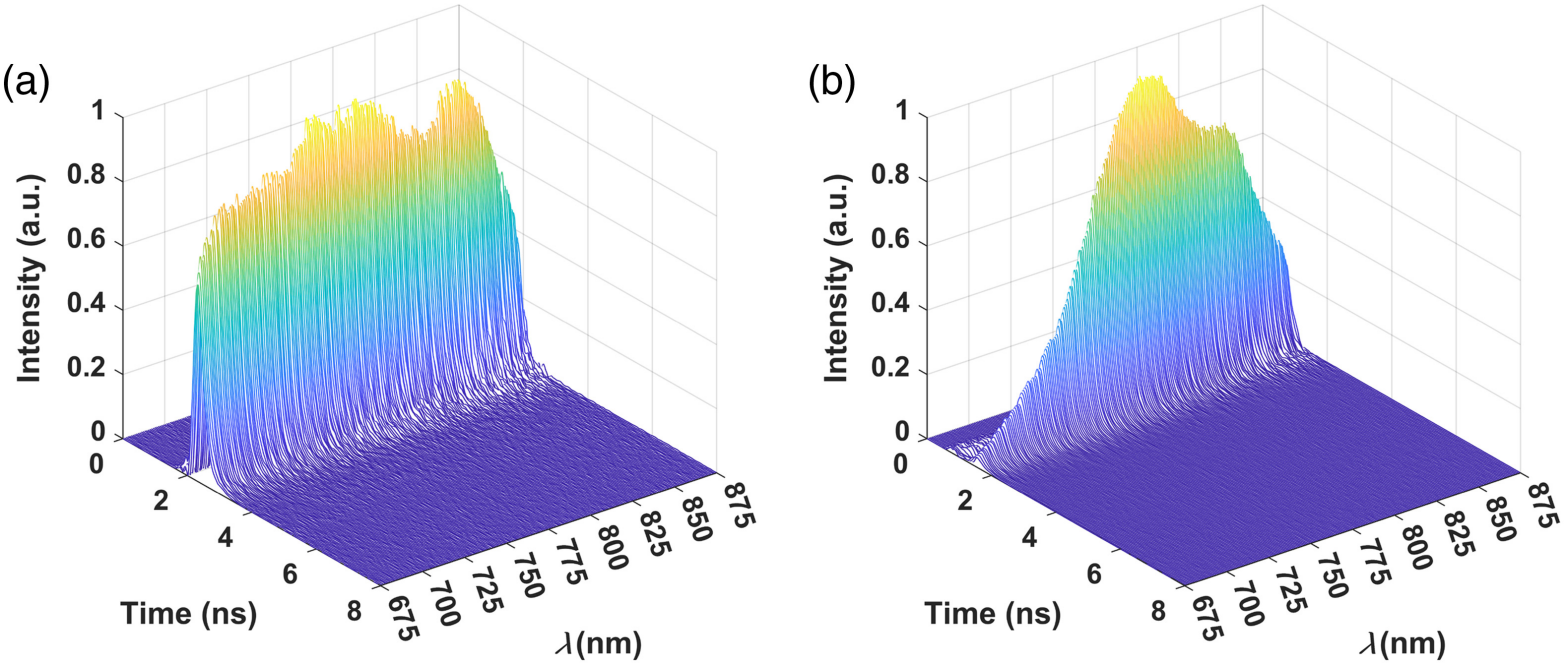
Three-dimensional plots of the uncompressed HyperTRCSS IRF using (a) the YSL laser and (b) the NKT laser across the spectral range of 675 to 875 nm.

The data acquisition scheme is detailed in Ref. 55, with modifications described in Ref. 56. Briefly, the DMD is used to implement a differential Hadamard single-pixel sampling strategy,^57^ where binary patterns are displayed sequentially on the DMD to reflect different sections of the spectrum toward the detector. If the number of Hadamard sampling patterns is equal to the number of points in the desired spectrum, the full spectrum can be recovered through a simple inversion. However, NIR spectra acquired from tissue can be sparsely represented in the spectral domain; therefore, a CS approach can be applied to reconstruct the spectrum from fewer sampling patterns.^55^ We previously demonstrated that there are no differences between estimations of changes in chromophore concentrations obtained from uncompressed and 90% compressed acquisitions.^56^ Therefore, the data were sampled using a 90% compression rate to reduce the acquisition time for a full spectrum of distributions of time-of-flight (DTOFs) to 20 s, corresponding to an effective sampling rate of 0.05 Hz. A DTOF collection time of 0.3 s was used along with a 0.05 s pause to ensure synchronicity between the DMD patterns and DTOF measurements. Note that DTOFs acquired outside of the 675 to 875 nm range had very low signal-to-noise ratio (SNR) and therefore were excluded from further analysis; the resulting spectra had 170 points and a resolution of 5.8 nm.^56^ Further details regarding the spectral calibration procedure are included in the Supplementary Material for reference. Figure 2 shows the uncompressed HyperTRCSS instrument response functions (IRFs) for both the YSL and the NKT laser. Note that IRFs were measured in a transmission configuration by placing a thin piece of white paper between the emission and detection probes. The pulse widths of the YSL and NKT lasers measured with our detection system were 556 and 547 ps, respectively. Both lasers were deemed temporally and spectrally stable following a 1 h warm-up period (see Supplementary Material). The maximum laser power was 0.26 mW, which is within the maximum permissible exposure guidelines for skin set by the American National Standard Institute (ANSI; 19.24 mW).^58^

### 2.2 Phantom Experiments

Blood-yeast liquid phantoms have been used to assess the ability of NIRS systems to estimate changes in Hb and HbO^50^^,^^59^^–^^61^ and even oxCCO.^56^^,^^62^ Here, we demonstrate HyperTRCSS monitoring of Hb, HbO, and oxCCO changes in homogeneous blood-yeast liquid phantoms with reference CW-NIRS measurements acquired concomitantly by a commercial spectrometer (Maya2000 Pro, Ocean Insight). We selected CW-NIRS as a reference because the current “gold standard” for oxCCO monitoring is CW measurement in 1-nm intervals across the wavelength range 780 to 900 nm.^16^^,^^20^^–^^23^ We subsequently demonstrate the sensitivity of HyperTRCSS to Hb, HbO, and oxCCO changes in the bottom layer of two-layer blood-yeast phantoms.

#### 2.2.1 Homogeneous phantoms

A homogeneous phantom experiment was conducted with each of the two supercontinuum lasers to demonstrate reproducibility. The phantom composition was based on methods reported in the literature.^50^^,^^59^^–^^62^ Each phantom consisted of an opaque PVC container (20×20×20  cm, L×W×H) filled with an Intralipid solution (168 mL of Intralipid-20% diluted to 0.8% through the addition of 2 L of phosphate-buffered saline and 2 L of distilled water) and whole animal blood. The volume of blood added in the first experiment was 20 mL and the resulting total hemoglobin concentration in the phantom, following dilution, was 7.5  μM. For the second experiment, the amount of Intralipid, phosphate-buffered saline, and distilled water was the same as in the first set of measurements; however, the volume of blood added was 10 mL and the estimated total hemoglobin concentration in the phantom was 6.4  μM. Note that 24.35 mL of sodium-bicarbonate buffer (SBB) was added in both experiments to maintain physiological pH (7.4) during deoxygenation/reoxygenation. During the experiments, phantom homogeneity was maintained by stirring with a magnetic stir bar, and a hot plate was used to maintain temperature at normal physiological level (37.5°C).

**Fig. 3:**
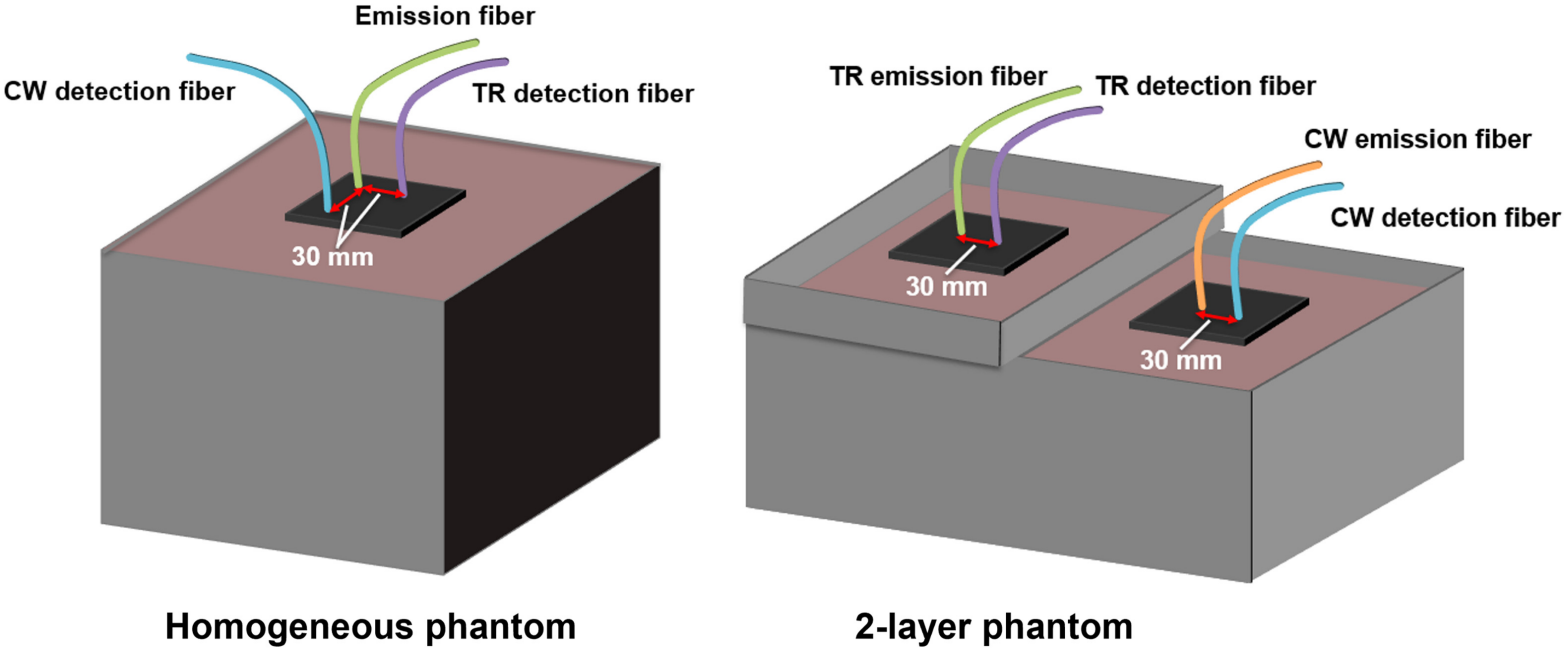
Schematic of homogeneous and two-layer phantom containers with probe configurations. All source–detector separations were 30 mm.

Following a 1 h system warm-up period, the emission and detection probes were placed in the center of the phantom as shown in Fig. 3. Probe tips were slightly submerged to ensure good contact with the phantom despite motion at the surface of the liquid during stirring.^62^ To minimize gas exchange with the ambient air, the top of the phantom was sealed with plastic wrap.^62^ Throughout the experiment, TR and CW acquisitions were taken concomitantly every 20 s. Further, an oxygen sensor (Foxy-AL300, Ocean Insight) was placed in the middle of the phantoms to monitor the partial pressure of oxygen in the solution (pO2) every 20 s.

The first experiment was performed with the YSL laser, with baseline measurements acquired for 8 min. The phantom was deoxygenated over 14 min by 4 g of baker’s yeast activated in 4 mL of 50% glucose and 2 mL of SBB. Following the deoxygenation, oxygen gas from an industrial air tank was bubbled into the solution through an air stone to reoxygenate the hemoglobin. The second homogeneous phantom experiment was conducted using the NKT laser. Baseline measurements were acquired for 13 min and the phantom was deoxygenated over 30 min. In this repetition, the amounts of baker’s yeast and glucose/SBB solution were reduced by half to simulate a slower rate of deoxygenation. For each experiment, a compressed acquisition of the IRF was taken before the phantom measurements by placing a thin piece of white paper between the emission and detection probes in a transmission configuration.

#### 2.2.2 Two-layer phantoms

The two-layer phantom experiment was also performed twice—once with each supercontinuum laser. The phantom container was constructed from opaque PVC and contained a bottom layer compartment (27×15×11  cm, L×W×H), which was separated from a shallow top layer compartment (13×14×3  cm, L×W×H) by a transparent plastic film base; compartments were arranged so that both the main basin and the top layer were accessible throughout the experiment (Fig. 3). A solution made up of 2 L of PBS, 1.73 L of distilled water, 157 mL of Intralipid-20%, 22.70 mL of SBB, and 10 mL of whole animal blood was stirred to homogeneity in the main basin. The resulting concentration of Intralipid was 0.8%, and the estimated total hemoglobin concentrations after dilution were 6.6 and 6.9  μM for the first and second experiments, respectively. After stirring for 30 min, part of the solution was transferred via a syringe from the main basin into the top compartment until the top liquid layer reached a height of 10 mm. The liquid in the main basin was stirred throughout the experiment.

Figure 3 shows the experimental setup with the TR and CW probes on the top and bottom phantom layers, respectively. Note that the TR device was warmed up for 1 h prior to probe placement, and—since the TR emission could not simultaneously serve as the CW light source—a halogen lamp (HL-2000-HP-FHSA, Ocean Insight) was coupled to a multimode fiber bundle for CW illumination. The oxygen sensor was placed in the bottom layer compartment to monitor the pO2. All measurements were acquired concomitantly every 20 s.

The first experiment was conducted with the YSL laser. Measurements were acquired for 8 min (baseline) before deoxygenation of the solution in the bottom compartment by 2 g of yeast dissolved in 2 mL of 50% glucose and 1 mL of SBB. No yeast was added to the top layer compartment to mimic the low mitochondrial concentration in the ECL relative to the brain.^25^^,^^63^ The bottom layer was left to deoxygenate for 28 min before reoxygenation was initiated by bubbling oxygen gas through the air stone. The NKT laser was used for the second experiment, and the same procedure for deoxygenation/reoxygenation was followed after 10 min of baseline acquisition. Before each experiment, a compressed IRF measurement and a reference spectrum of the CW light source were acquired through a piece of white paper in a transmission configuration.

### 2.3 Data Processing and Analysis

#### 2.3.1 Processing of the TR and CW data

The TR data were reconstructed and processed using the pipeline described in Ref. 56, and each acquisition provided a 170-point spectrum spanning 675 to 875 nm. The CW data were processed by first subtracting the mean dark signal from each spectrum. The change in attenuation, ΔA(λ), relative to the baseline was then calculated for each acquisition using Eq. (1):

$$\Delta A(\lambda )=-\ln(\frac{I(T,\lambda )}{I(0,\lambda )}),$$ (1)

where I(T,λ) is the spectrum at time point T and I(0,λ) is the average spectrum at baseline.

#### 2.3.2 TR data analysis

The TR data were analyzed using the late-photon fitting approach^40^^,^^64^^,^^65^ to estimate the absorption coefficients in both the homogeneous and two-layer phantoms because it is simpler and faster to implement than computationally intense methods (e.g., fitting to a solution to the diffusion approximation^35^^,^^66^) while offering good depth-sensitivity.^38^^,^^40^^,^^41^ In this method, the tails of the DTOFs are used to estimate the wavelength-dependent absorption coefficient, $\mu_{a}(\lambda )$, through Eq. (2):

$$\mu_{a}(\lambda )=-\frac{m(\lambda )}{c/n},$$ (2)

where m is the asymptotic slope of the natural logarithm of the DTOF, c is the speed of light in a vacuum, and n is the refractive index of the phantom (assumed to be 1.33^60^). The definition of the tail of the DTOF varies in the literature; some suggest that the slope can be fit after the intensity of the DTOF falls below 50% to the right of the peak intensity,^64^ whereas others suggest using later windows (10% to 2%^67^ or 5% to 1%^68^^,^^69^) to ensure that the asymptotic assumption is met.^65^ Importantly, using a later window comes at the cost of SNR since the number of detected photons is lower.^70^ Due to current system limitations, the SNR in the 5% to 1% window was quite low, with the mean SNR across wavelength falling as low as 6±3. The SNR between 20% and 5% was also low (8±4 for one of the phantom experiments); thus, we deemed any window below 20% to be unusable in the analysis. Nevertheless, the data had sufficiently good SNR in the range of 50% to 20% to consistently provide a robust estimate of the slope (typical mean SNR across wavelength was 21±9). $\Delta \mu_{a}(\lambda )$ was computed by subtracting the baseline $\mu_{a}(\lambda )$ and then fit to Eq. (3) using a least-squares minimization approach^56^ to estimate changes in chromophore concentrations, i.e., ΔHb, ΔHbO, and ΔoxCCO:

$$\Delta \mu_{a}(\lambda )=\Delta \mathrm{Hb}\varepsilon_{\mathrm{Hb}}(\lambda )+\Delta \mathrm{HbO}\varepsilon_{\mathrm{HbO}}(\lambda )+\Delta \mathrm{oxCCO}\varepsilon_{\mathrm{ox}\text{-redCCO}}(\lambda ),$$ (3)

where $\varepsilon_{\mathrm{Hb}}(\lambda )$ and $\varepsilon_{\mathrm{HbO}}(\lambda )$ are the hemoglobin specific extinction coefficients, and $\varepsilon_{\mathrm{ox}\text{-redCCO}}(\lambda )$ is the difference spectrum of the specific extinction coefficients for oxCCO and redCCO.^71^ We set ΔHbO=−ΔHb because the total hemoglobin was constant in the phantom throughout deoxygenation and reoxygenation. Therefore, there were only two fitting parameters: ΔHb and ΔoxCCO. As well, the water content of the phantom was assumed to be constant throughout the experiment.

#### 2.3.3 CW data analysis

For the CW data, we implemented a multi-step spectral derivative analysis using the differential form of the modified Beer–Lambert law:^72^

$$\Delta A(\lambda )=\Delta \mu_{a}(\lambda )L(\lambda ),$$ (4)

where $\Delta \mu_{a}(\lambda )$ is as defined in Eq. (3) and L(λ) is the wavelength-dependent optical pathlength. Note that cross talk is minimized in spectral derivative approaches^73^^,^^74^ relative to traditional methods, which fit for all the chromophores at once. In the first step, we fit the second derivative of the measured ΔA(λ) with the second derivative of Eq. (4) between 720 and 780 nm to recover ΔHb with minimal cross talk since the second-derivatives of $\varepsilon_{\mathrm{oxCCO}}$ and $\varepsilon_{\mathrm{HbO}}$ are featureless in this spectral window.^73^ Note that the ΔA(λ) were smoothed using a moving average filter before taking spectral derivatives. The second step fixed ΔHb, assumed ΔHbO=−ΔHb, and fit Eq. (4) between 815 and 845 nm with ΔoxCCO as the only fitting parameter. Note that 815 to 845 nm was selected as it is centered around the 830 nm oxCCO peak.^16^

#### 2.3.4 Monte Carlo simulations

Mesh-based MC simulations were conducted in MMCLAB^75^ to (a) evaluate and refine the data analysis methods before their application to experimental data, (b) assess the impact of fitting 50% to 20% instead of 5% to 1% of the DTOF tail in the TR analysis, and (c) provide a point of comparison for the experimental results. To mimic the geometry of the two-layer phantoms, we generated a mesh of a two-layer box (10  cm×10  cm×1  cm top layer, 10  cm×10  cm×10  cm bottom layer; L×W×H) using the BlenderPhotonics pipeline.^76^ The source and detector were placed on the center of the top layer surface and were separated by 30 mm. Eleven levels of oxygenation were simulated in the bottom layer of the phantom while the top layer absorption coefficients remained constant. A detailed description of the optical properties assigned to each layer is provided in the Supplementary Material. In addition, we conducted homogeneous phantom simulations where we simply set the optical properties of the top layer to those of the bottom layer. To ensure realistic photon statistics and high SNR at each wavelength, $10^{7}$ and $10^{8}$ photons with random seeds were used in the homogeneous and the two-layer phantom simulations, respectively.

#### 2.3.5 Analysis of the simulated data

To evaluate the TR analysis, each of the temporal point spread functions (TPSFs) generated in the MC simulations was first convolved with an experimental IRF to generate DTOFs. Since two different light sources were used in the experiments, each TPSF was convolved with an IRF acquired with the YSL and the NKT laser, resulting in two sets of DTOF data for each simulated TPSF. Each DTOF dataset was then analyzed with the TR late-photon fitting analysis described in Sec. 2.3.2, using the data from the first simulation (i.e., simulation #1) as the baseline acquisition. For the definition of the tail, we used 5% to 1% in addition to 50% to 20% to estimate how much accuracy would have been gained if the experimental data had high enough SNR to allow for the use of the 5% to 1% window. We then fit the $\Delta \mu_{a}$ as described in Sec. 2.3.2 between 680 and 840 nm, which we determined to be the optimal range for recovering ΔHb and ΔoxCCO from the HyperTRCSS data through systematic testing (see Supplementary Material). For the CW analysis, we temporally integrated the TPSFs, which resulted in 11 spectra. The change in attenuation at each deoxygenation level was calculated with respect to the first simulation, which was assumed to be the baseline, and the changes were analyzed using the CW analysis described in Sec. 2.3.3. The optical pathlength was calculated from the mean TOFs of the TPSFs. The percent error from the ground truth was calculated for each analysis method (TR analysis with a 50% to 20% fitting window, TR analysis with a 5% to 1% fitting window, and CW analysis).

#### 2.3.6 Analysis of the experimental data

Changes in chromophore concentrations in the homogeneous and two-layer phantom experiments were recovered from the HyperTRCSS data using the TR data analysis described in Sec. 2.3.2, with the 50% to 20% definition of the DTOF tail and the optimized wavelength range (680 to 840 nm) from Sec. 2.3.5 for fitting the recovered $\Delta \mu_{a}$. Changes in chromophore concentration from the CW measures in the homogeneous phantoms were estimated using the CW data analysis described in Sec. 2.3.3. The wavelength-dependent pathlength at each time point was derived from the concomitantly acquired TR data using the mean TOF for each wavelength. For the two-layer experiments, we could not use the TR data directly to estimate the pathlength because the TR and CW systems were not probing the same compartment of the phantom. Instead of taking the pathlength directly from the TR data as an estimate, we fit the second derivative of the CW reflectance at each time point to the second derivative of the water absorption spectrum^77^ (water fraction was assumed to be 0.99 given the known composition of the phantom) and corrected for wavelength dependence using the time-point-specific spectral shape of the TR pathlength. It is likely that the shape of the TR pathlength did not change as much as that of the CW pathlength during deoxygenation/reoxygenation because the medium probed by CW-NIRS was more dynamic;^78^ however, this approach was more likely to yield accurate results than traditional methods^5^^,^^24^^,^^25^ that assume that the spectral shape of the pathlength is constant during deoxygenation and reoxygenation. For each experiment, the Spearman correlations (ρ) between the TR and CW results were calculated (α=0.05). The strength of the correlations was interpreted using standards outlined in Ref. 79.

## 3 Results

### 3.1 Simulation Results

**Fig. 4:**
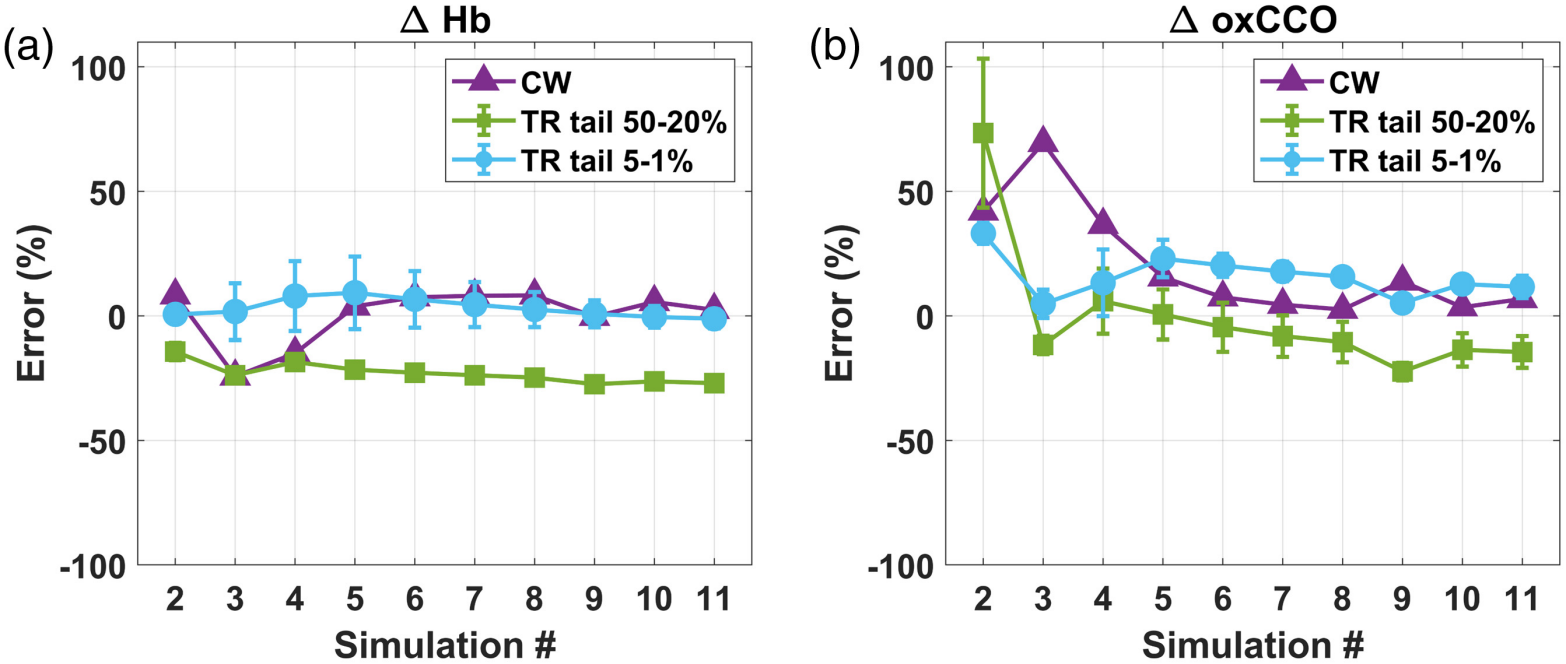
Error (%) of each of the three analysis methods (CW, purple; TR late-photon fitting between 50% and 20% of the DTOF tail, green; TR late-photon fitting between 5% and 1% of the DTOF tail, blue) for estimating (a) ΔHb and (b) ΔoxCCO in a homogeneous phantom model. The TR results are plotted as mean ± SD. Only simulations #2 to #11 are plotted here because changes in chromophore concentration were computed with respect to simulation #1.

Figure 4 displays the error of the different analyses when estimating changes in chromophore concentration for the homogeneous phantom simulations. Errors in the estimated changes in chromophore concentration are shown as percentages relative to the inputted changes. Since there were two sets of DTOF data (TPSFs convolved with the YSL IRF and the NKT IRF), the two sets of TR results were averaged for plotting. Since we assumed ΔHbO=−ΔHb in the analysis, only the errors for ΔHb and ΔoxCCO estimates are shown. Further, because the sign of the recovered ΔHb and ΔoxCCO was consistently the same as the sign of the ground truth ΔHb and ΔoxCCO, a negative error is interpreted as an underestimation of the changes in chromophore concentration and a positive error as overestimation.

For the homogeneous phantom simulations, the mean error across simulation # for ΔHb estimated by CW and TR tail-fitting between 5% and 1% was negligible (0.4±11% for CW and 3±4% for TR tail-fitting between 5% and 1%). However, TR tail-fitting between 50% and 20% consistently underestimated the ground truth ΔHb, with mean error of −23±4%. In all three analysis methods, the largest errors in ΔoxCCO recovery occurred in the first few simulations (#2 to #4), where the changes in oxCCO concentration were the smallest in absolute value. As changes in oxCCO concentration increased, there was a decrease in the errors for CW. The accuracy of ΔoxCCO estimation tended to be worse than ΔHb estimation. On average, for simulations #5 to #11, the mean error was 8±5% for CW, 15±6% for TR tail-fitting between 5% and 1%, and −10±7% for TR tail-fitting between 50% and 20%.

**Fig. 5:**
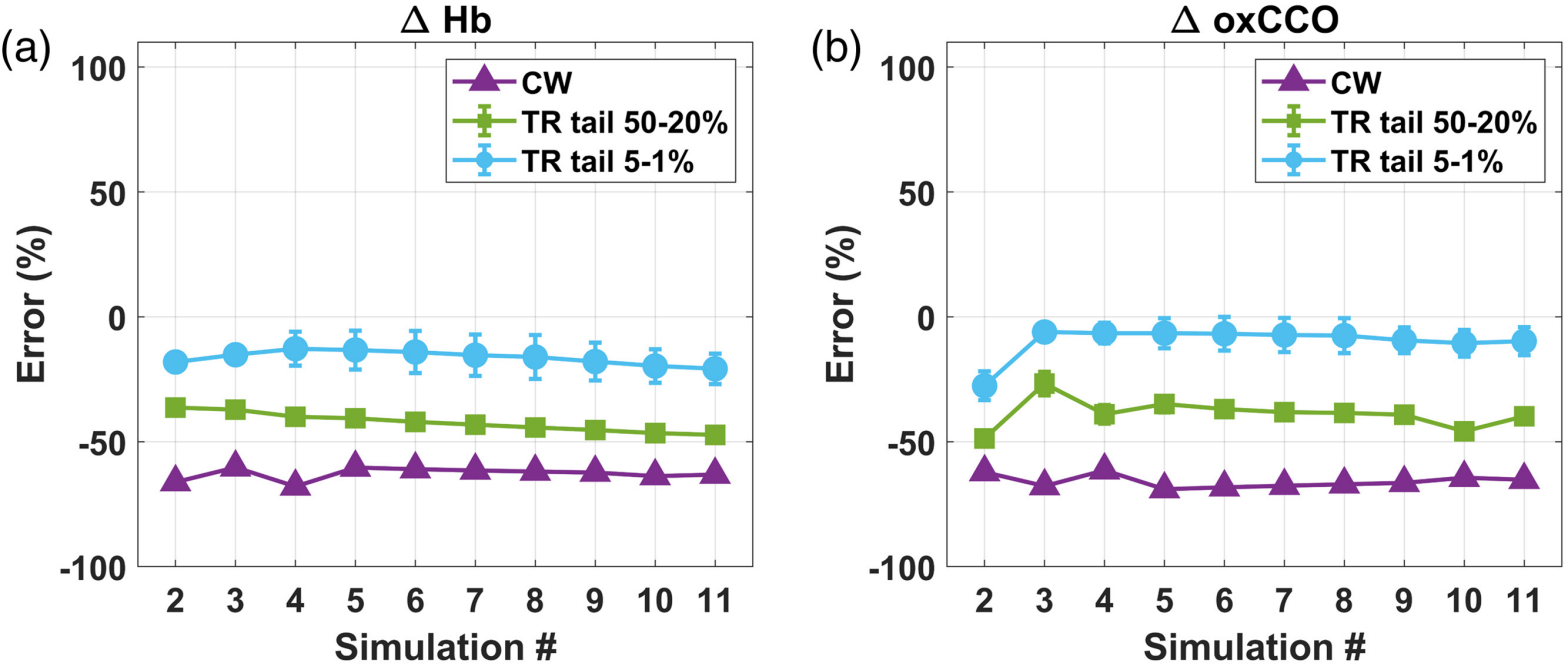
Comparison of percent error (%) for each of the three data analysis approaches (CW, purple; TR late-photon fitting between 50% and 20% of the DTOF tail, green; TR late-photon fitting between 5% and 1% of the DTOF tail, blue) for (a) ΔHb and (b) ΔoxCCO in a two-layer phantom model. The TR results are plotted as mean ± SD. Only simulations #2 to #11 are plotted here because chromophore concentration changes are with respect to simulation #1.

Figure 5 shows the errors for the same data analysis methods in the two-layer phantom simulations. In this case, the CW method had the largest error and underestimated the ground truth changes in chromophore concentration with error consistently greater than −60% for both ΔHb and ΔoxCCO (mean errors across simulation # were −63±3% and −66±3%, respectively). TR tail-fitting between 50% and 20% also underestimated chromophore concentration changes with errors in ΔHb steadily increasing with simulation # up to −47% for simulation #11 (mean error in ΔHb was −42±4%). Error in ΔoxCCO was on average −39±4%. As expected, TR tail-fitting between 5% and 1% performed the best, with error in ΔHb only reaching −21% for simulation #11 (mean error was −16±3%). Mean error in ΔoxCCO was −10±6%.

### 3.2 Experimental Results

**Fig. 6:**
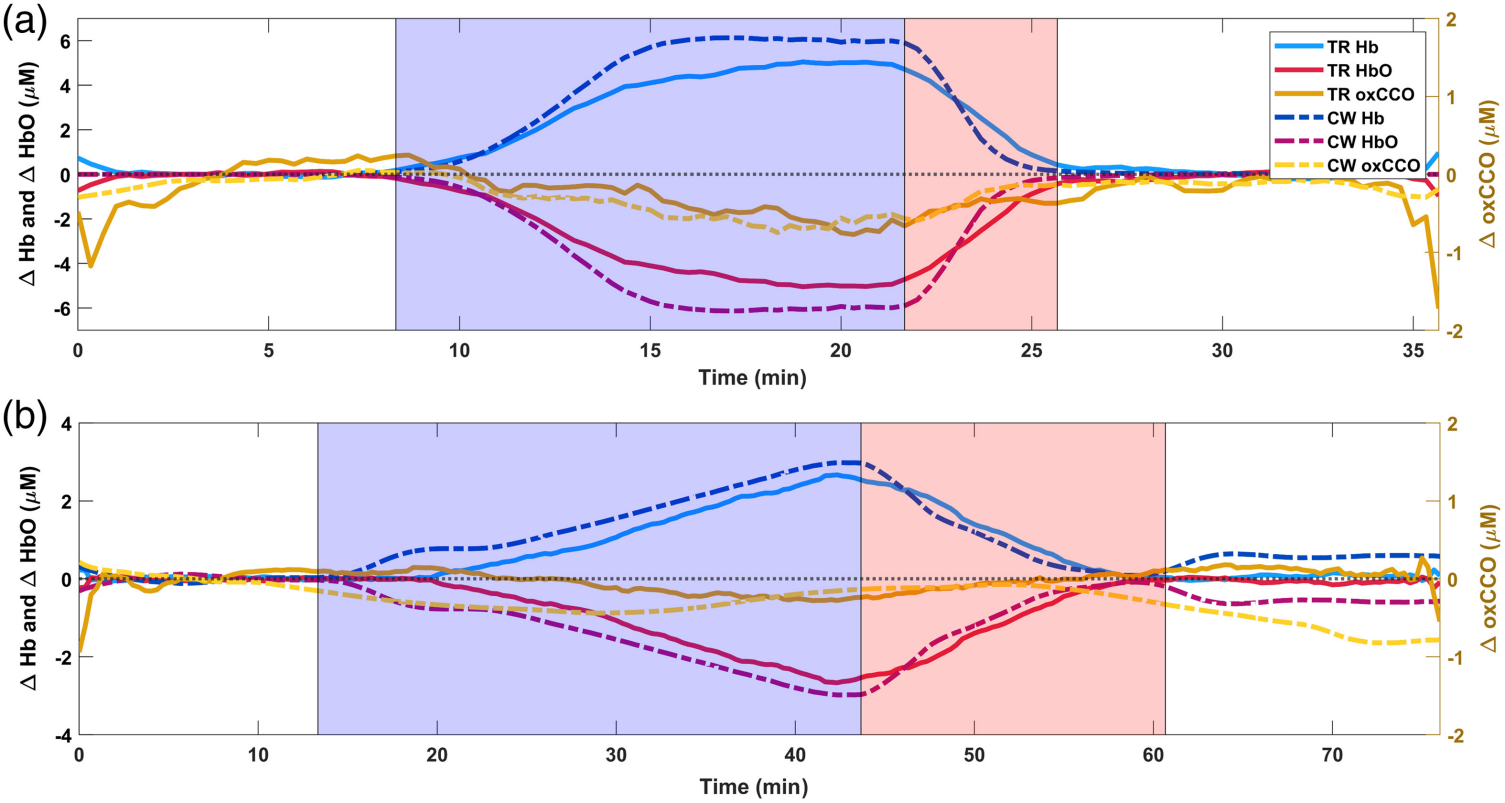
Hemoglobin and oxCCO time courses from repetitions (a) 1 and (b) 2 of the homogeneous phantom experiment. The deoxygenation and reoxygenation periods are indicated by the blue and red shaded regions, respectively. The pO2 reading on the oxygen probe was 0% during these periods. Changes in chromophore concentrations estimated from TR data are plotted with solid lines, and those obtained from CW data are plotted with dashed lines. The hemoglobin traces are plotted on the left y-axis, and oxCCO traces are plotted on the right y-axis. The black horizontal dotted line represents 0 change from baseline for all chromophores.

**Fig. 7:**
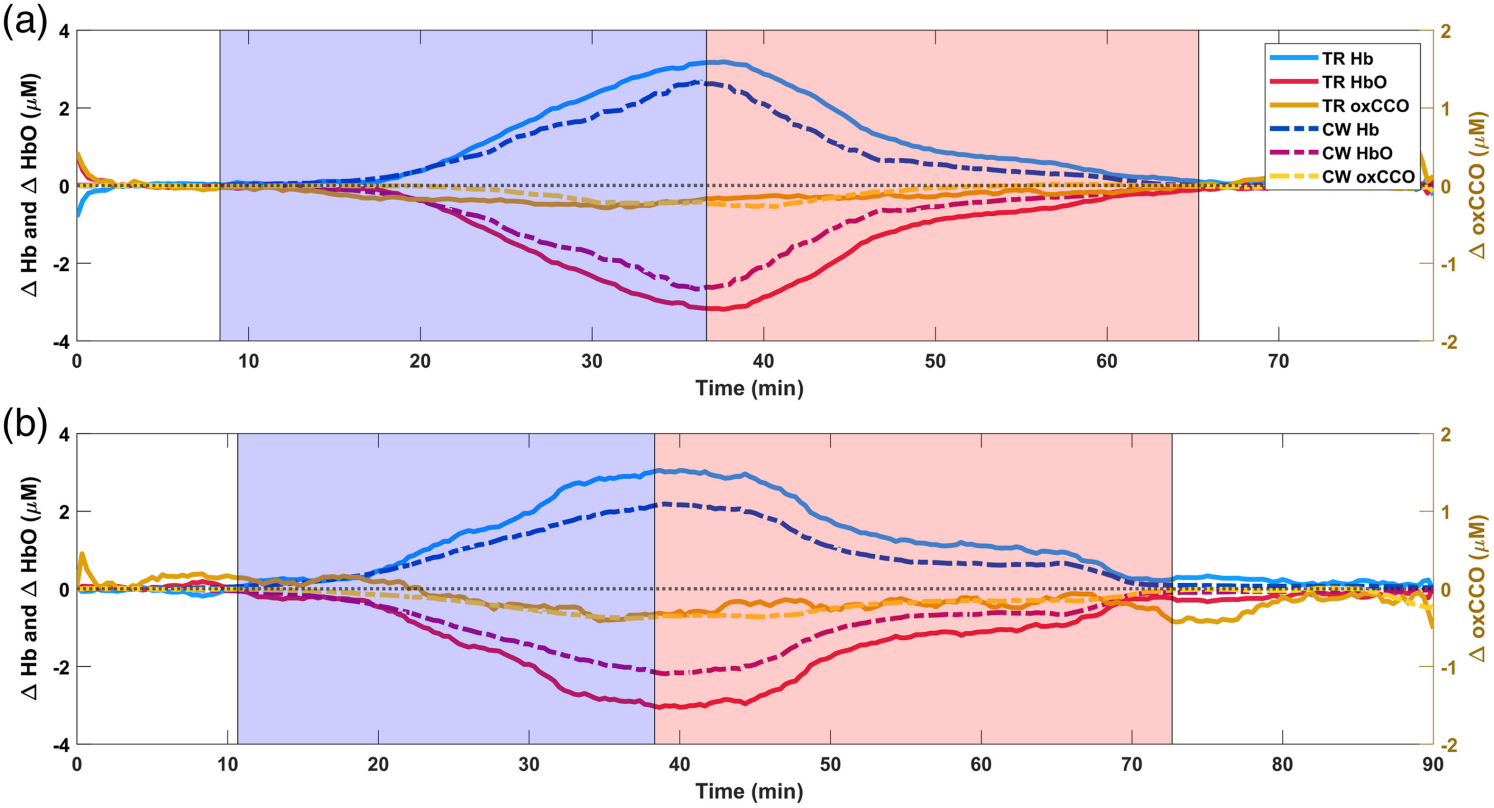
Hemoglobin and oxCCO time courses from repetitions (a) 1 and (b) 2 of the two-layer phantom experiments. The deoxygenation and reoxygenation periods are indicated by the blue and red shaded regions, respectively. The pO2 reading on the oxygen probe was 0% during these periods. Changes in chromophore concentrations estimated from TR data are plotted with solid lines, and those obtained from CW data are plotted with dashed lines. The hemoglobin traces are plotted on the left y-axis, and oxCCO traces are plotted on the right y-axis. The black horizontal dotted line represents 0 change from baseline for all chromophores.

Figure 6 shows the changes in chromophore concentrations recovered from the TR and CW data sets for the homogeneous phantom experiments. Figure 7 provides the same information but for the two-layer phantom experiments. The hemoglobin deoxygenation began when the pO2 reading on the oxygen probe reached 0%. In all the experiments, the concentration of Hb increased with a concomitant decrease in the concentration of HbO during the deoxygenation phase. This was accompanied by a decrease in oxCCO. At the start of reoxygenation, these trends inverted, and all concentrations returned to baseline as pO2 began to rise above 0%. The duration of deoxygenation and reoxygenation varied with the total hemoglobin in the phantom, amount of yeast added, the period of time before oxygen gas was bubbled into the solution, and the rate at which the oxygen bubbled through the air stone.

For repetition 1 of the homogeneous phantom experiment [Fig. 6(a)], the TR estimates of the hemoglobin changes were smaller than the CW estimates. The oxCCO estimates by TR and CW generally agreed well over time. The TR and CW results were generally consistent in terms of the direction and timing of the concentration changes, except during reoxygenation when the CW results showed faster hemoglobin reoxygenation than the TR results. These trends were similar during repetition 2 of the homogeneous phantom experiment [Fig. 6(b)], with the TR hemoglobin measurements underestimating the CW values. While the oxCCO results in repetition 2 generally agreed in terms of the magnitude of the estimated changes, the timing of those changes was quite different, unlike in repetition 1.

For the two-layer phantom experiments [Figs. 7(a) and 7(b)], unlike in the homogeneous phantoms, the TR estimates of hemoglobin concentration changes were larger than the CW estimates. The oxCCO estimates by TR and CW generally agreed well over time for both repetitions; however in repetition 2, the TR oxCCO trace appears to oscillate around the CW trace during reoxygenation and then dips down again after the end of reoxygenation between minutes 70 and 80. The Spearman correlation coefficients (ρ) for all experiments are presented in Table 1. Since ΔHbO=−ΔHb, only correlations between TR and CW for ΔHb and ΔoxCCO are shown. All correlations between TR and CW results were significant with p<0.001.

**Table 1:** Spearman correlation coefficients (ρ) between TR and CW results for each chromophore. n indicates the number of TR and CW acquisitions taken over the course of the experiment.

Phantom type	Homogeneous		Two-layer	
**Repetition #**	1	2	1	2
n	108	229	238	271
$\rho_{\Delta \mathrm{Hb}}$	0.881^a^	0.827^a^	0.962^a^	0.983^a^
$\rho_{\Delta \mathrm{oxCCO}}$	0.839^a^	−0.313^a^	0.522^a^	0.561^a^

a Indicates that the correlation is significant at the 0.001 level.

## 4 Discussion

Hyperspectral TR-NIRS can quantify tissue absorption and scattering across a broad spectral range with excellent depth sensitivity. However, practical implementation of the technology has been challenging owing to the inherent trade-off between spectral range and resolution.^44^^,^^48^^,^^50^ Further, systems that achieve high resolution over a wide spectral range often necessitate lengthy acquisition times, rendering them unsuitable for monitoring dynamic physiology.^43^^,^^45^^,^^47^ This work presents the implementation of a hyperspectral TR spectrometer that harnesses CS to minimize acquisition time while maintaining wide spectral range and high spectral resolution.

The unique design of the spectrometer enables acquisition of 170-point spectra spanning 675 to 875 nm with high spectral resolution (5.8 nm), in just 20 s. Although scanning-style hyperspectral TR spectrometers are capable of higher resolution (1 nm), acquisition times for these systems scale linearly with the spectral range covered. At acquisition rates of 0.8 to 1 s per wavelength,^45^^,^^47^ these scanning spectrometers would require over 2 min to acquire a 170-point spectrum, which is too slow for monitoring dynamic physiologic systems. Hyperspectral TR spectrometers with parallel detection can acquire spectra faster than our system (≤1  s);^44^^,^^48^^,^^50^ however, to cover an equivalent spectral range, these systems would have to acquire more wavelengths per channel, which would lead to much lower spectral resolution (12.5 to 20 nm per channel). While our system is also limited by the trade-off between range and resolution, it can achieve twice the spectral resolution of these other systems while spanning the same range. Further, the spectra acquired by parallel-detecting spectrometers have a limited number of points, equal to the number of channels (often 16), which precludes the use of derivative spectroscopy techniques that minimize cross talk.^73^^,^^74^ Although derivative spectroscopy was not applied to the TR data in this work, the high spectral sampling of our system could enable such analysis in the future. Future work could also further reduce the acquisition time of our spectrometer by applying a pulse pile-up correction algorithm^80^ so that higher photon count rates and shorter DTOF collection times can be used.^81^ Notably, an increase in photon count rate from 1 to 5% of the laser pulse rate should result in a fivefold decrease in the overall acquisition time and would bring us closer to the goal of real-time monitoring.

The homogeneous phantom experiments confirmed the sensitivity of our system to changes in Hb, HbO, and oxCCO concentrations. In all repetitions, the expected changes in TR and CW measures occurred when the pO2 dropped below 0% (HbO and oxCCO decreasing while Hb increased), and when the oxygen gas was bubbled into solution (HbO and oxCCO increasing while Hb decreased). The fact that this was true for all repetitions, for both TR and CW instruments, suggests that the general trends observed in Hb, HbO, and oxCCO were real and not spurious. The magnitude of the TR estimates of Hb and HbO changes was smaller than the CW estimates, which was consistent with the predictions from the simulation results and with the results of a similar study performed with multi-wavelength TR-NIRS.^62^ Fitting the tails of the simulated DTOFs between 50% and 20% of the peak intensity resulted in underestimation of the ground truth by ∼23%, whereas CW recovered the ground truth accurately. The accuracy of CW was generally consistent for all simulations, except for some early points (#3 and #4), where a small absolute error may be inflated when expressed as a percentage of a small change in the input concentration. As expected, using the 5% to 1% tail-fitting window provided accurate results as well. Given that the simulated medium was homogeneous and that all photons in the DTOF probed the same tissue type, the increase in accuracy when using the 5% to 1% tail-fitting window on the simulated data suggests that the asymptotic assumption of Eq. (2) was not met when using the 50% to 20% tail-fitting window.^65^ Although the 5% to 1% window could not be used in our experiments due to the current limitations of the spectrometer, modifications to improve SNR should enable the use of this window in the future. Despite the underestimation by the 50% to 20% tail-fitting, the high positive correlations between TR and CW measures suggest that the instruments were measuring the same physical changes in the phantoms (Table 1).

The simulations also support the observed agreement between the TR and CW oxCCO measures during deoxygenation and reoxygenation, with the exception of the first 20 min of deoxygenation in repetition 2. A possible explanation for this discrepancy was instability in the spectral shape of the light source. Although the stability tests suggested that the NKT laser was stable after 1 h of warm-up, the non-zero ΔA(λ) after reoxygenation (when the pO2 >0, representing a return to baseline) provided evidence of spectral instability (see Fig. S3 in the Supplementary Material). The suspected change in the spectral shape of the light source did not impact the TR analysis since the absolute $\mu_{a}(\lambda )$ at each time point was estimated before relative changes were computed. However, since the CW analysis directly relates the ΔA(λ) to $\Delta \mu_{a}(\lambda )$, changes in the spectral shape of the light source could have been erroneously attributed to changes in chromophore concentrations during the experiment. The changing spectral shape of the light source could also explain the deviation in CW measures from baseline following the completion of reoxygenation. Given the high qualitative agreement and high positive correlation between TR and CW results for oxCCO in repetition 1 of the homogeneous phantom experiment, we suspect that the TR oxCCO results in repetition 2 are more representative of the true oxCCO changes in the phantom than the CW results. The fact that the TR analysis was unaffected by the light source spectral instability highlights another advantage of its use over CW methods.

There was also evidence of a wavelength-independent increase in the intensity of the YSL laser in repetition 1 of the homogeneous phantom experiment (see Fig. S3 in the Supplementary Material), but this was corrected for by fitting an additional wavelength-independent amplitude term in both steps of the CW analysis. Unfortunately, the changes in the NKT laser spectral shape could not be corrected for in repetition 2. For future experiments, we plan to use the YSL laser given its superior spectral stability. Alternatively, a fraction of the NKT laser beam could be deflected to a reference CW spectrometer to monitor its spectral shape, allowing for correction in the CW analysis. Despite the challenges caused by the spectral instability of the supercontinuum light sources for the CW measurements, the homogeneous phantom experiments confirmed that the TR spectrometer is sensitive to real changes in Hb, HbO, and oxCCO concentrations. In addition, the simulation results supported our experimental findings, giving us greater confidence in the accuracy of our results.

The main objective of this work was to test the hypothesis that the hyperspectral TR-NIRS device is sensitive to changes in blood oxygenation and oxCCO in deep tissue (>10  mm) through a thick superficial layer. By changing the oxygenation of the bottom layer of the phantoms while keeping the 10 mm top layer at a static oxygenation level, we anticipated that surface contamination, if present, would result in TR measures underestimating the true changes in the bottom layer. The experimental results show that the TR estimates of ΔHb and ΔHbO were actually larger than the estimates obtained from CW, whereas ΔoxCCO estimates between the two modalities generally agreed well. The two-layer phantom simulations suggested that while TR tail-fitting would substantially minimize surface contamination relative to CW analysis, it would not eradicate it completely. The use of the 50% to 20% window was also expected to lead to underestimation of the true changes. Given the high accuracy of the CW measures in the homogeneous phantom simulations, TR measures were anticipated to underestimate the CW measures taken directly from the bottom layer. The reason for the discrepancy between the predictions from the simulations and the experimental results is likely related to the inherent difficulty in estimating the optical pathlength in the CW data analysis. The spectral shape of the optical pathlength varies with absorption of light by chromophores, with greater absorption reducing the probability of light with longer pathlengths reaching the detector.^13^ In the simulations, the true optical pathlength was known; however, in the two-layer phantom experiments, the optical pathlength was estimated using the water peak in the second derivative of the CW reflectance data and corrected with the time-point-specific spectral shape of the TR pathlength. Since the TR pathlength was obtained using mean TOF, which is susceptible to surface contamination,^27^^,^^28^ it is unlikely that the spectral shape of the TR pathlength changed as much as the spectral shape of the CW pathlength with oxygenation changes in the bottom layer. By not fully accounting for the changes in pathlength with changes in absorption, changes in chromophore concentrations may be underestimated in the CW analysis as a result of cross talk with the pathlength.^13^^,^^82^ Estimation of changes in oxCCO is especially sensitive to inaccuracies in the pathlength, given its small concentration changes relative to those of hemoglobin.^78^^,^^82^ Although there are methods to estimate the optical pathlength’s wavelength dependence using CW data alone,^82^ these approaches assume that changes in attenuation are predominately caused by changes in hemoglobin and neglect the contribution of oxCCO. The challenges of accurately quantifying optical pathlength from CW data alone further support the use of TR methods over CW for accurate quantification of changes in chromophore concentration.

Further, there were oscillations followed by a sudden drop in TR oxCCO at the end of the reoxygenation period in repetition 2 of the two-layer phantom experiment [Fig. 7(b)]. The exact reasons for the sudden drop are unclear, but we suspect that the oscillations may be related to challenges in keeping the rate of oxygen bubbling consistent. Normally, the rate of oxygen bubbling is sufficient for the oxygenation of the phantom to rise steeply and consistently, but in this experiment, the rate of oxygen bubbling often tapered and the gas regulator needed to be adjusted.

The two-layer phantom simulations strongly support the use of TR methods to minimize superficial layer contamination. As anticipated, the CW approach performed the worst when estimating ΔHb and ΔoxCCO in the bottom layer from the surface of the two-layer phantoms, substantially underestimating the changes in chromophore concentrations. This was anticipated given that the probes were placed atop a 10 mm thick static surface layer and previous studies have shown that the CW-NIRS signal in the adult head is dominated by ECL contributions.^27^^–^^29^ Since the concentrations changed only in the bottom layer while the top layer remained static, it was expected that CW would underestimate the changes in the bottom layer due to contamination by the top layer (partial volume effect).^72^ While CW sensitivity to the brain can be improved by using longer source–detector separations, this also comes with a loss in SNR;^83^ thus, the need to maintain adequate SNR imposes an inherent limit on the sensitivity of CW-NIRS to changes in deep tissue. In contrast, in TR-NIRS, the depth information is encoded in the arrival times of the photons with a marginal dependence of depth sensitivity on source–detector distance.^70^^,^^84^ By analyzing photons with later arrival times, one may improve sensitivity to deeper tissue layers even at short source–detector distances.^84^^,^^85^ Thus, we expected the TR tail-fitting between 50% and 20% to be less sensitive than the 5% to 1% window to changes in the deep layer of the simulated two-layer phantom. Indeed, the TR tail-fitting between 50% and 20% performed better than CW but worse than fitting between 5% and 1%. The homogeneous phantom results suggest that some of the error in the estimates by TR tail-fitting between 50% and 20% may be associated with not meeting the asymptotic assumption of Eq. (2). Nevertheless, the underestimation of the true changes in the two-layer phantom was even larger than what was observed in the homogeneous phantom, suggesting contamination from the top layer. Although the 50% to 20% tail-fitting window was more sensitive to the deeper-lying bottom layer than CW, using the later-arriving photons in the 5% to 1% window will be the preferred technique moving forward. Not only was the asymptotic assumption met with the 5% to 1% window, but a larger fraction of the photons in this window probed the deeper-lying bottom layer compared to the 50% to 20% window. Of the three methods tested on the simulated data, TR tail-fitting between 5% and 1% provided the most accurate estimates of the changes in ΔHb and ΔoxCCO. Based on these findings, future work will focus on modifying the system to improve the SNR of the experimental measurements so that the 5% to 1% window can be used in the tail-fitting analysis.

Despite the fact that the TR estimates of the changes in chromophore concentrations in the two-layer phantoms were likely underestimates of the true changes due to the use of the 50% to 20% window, HyperTRCSS was still able to recover changes in blood oxygenation and metabolism better than CW—the current “gold standard.” TR and CW measures of ΔHb and ΔHbO were strongly correlated, and even measures of ΔoxCCO, which were noisier, were moderately correlated, suggesting that the instruments were generally sensitive to the same trends. The two-layer phantom experiments and simulations support our hypothesis that the hyperspectral TR-NIRS device could monitor changes in oxCCO and blood oxygenation in deep layers (>10  mm) through a 10 mm thick static superficial layer. This result provides strong motivation for continued development of hyperspectral TR systems.

While the results of this study were highly encouraging, there were some limitations. One limitation of the study design is that we did not acquire CW data from the surface of the two-layer phantoms to experimentally demonstrate the relative reduction in top layer contamination of TR versus CW. Although the DTOFs could have been temporally integrated to calculate ΔA(λ), the resulting ΔA(λ) were too noisy to apply the CW spectral derivative analysis. Nevertheless, the simulation data clearly demonstrate that TR reduces superficial layer contamination compared to CW and we intend to experimentally validate this finding in future work. Further, the low SNR of the DTOF tails limited the effectiveness of the tail-fitting analysis on the experimental TR data. SNR could be improved by keeping the DTOF collection time the same while increasing the laser power to just below the ANSI limit. This would increase the photon count rate above 1% of the laser repetition rate, but as mentioned earlier a pulse pile-up correction could be applied to mitigate any negative effects.^80^^,^^81^ Alternatively, the hybrid PMT detector could be replaced with a single photon avalanche detector (SPAD) array to increase the number of TCSPC events acquired per collection period.^86^ Depending on the size of the SPAD array, the increase in SNR could be enough for the collection time per DTOF to be reduced even further to allow for faster sampling. Another limitation is that the concentrations of hemoglobin used in the phantoms were lower than what is typical in adult humans (50 to 80  μM ^87^). Thus, further validation in tissue-mimicking phantoms with higher hemoglobin concentrations or *in vivo* is needed before conclusions can be made about the sensitivity of the system to adult cerebral oxCCO. As well, the effects of physiological noise in the ECL remain to be investigated. In addition, a single ECL thickness of 10 mm was used for both the simulations and phantom experiments, which is a conservative estimate of adult ECL thickness. Future work should evaluate the impact of increasing the ECL thickness (e.g., up to 15 mm) on TR sensitivity to the deep layer. Finally, our work did not explore analysis with data from multiple source–detector distances. A second source–detector pair could be added in future work, which would allow for the use of more sophisticated methods of TR data analysis, such as subtraction-based approaches^88^ or approaches based on the solution to the diffusion equation for two-layer media.^36^^,^^69^

Future work will include *in vivo* testing of HyperTRCSS; however, before that, the SNR limitations identified in this study must be addressed. Potential solutions include replacing the current hybrid PMT detector with a SPAD array and increasing the photon count rate above 1% of the laser repetition rate in combination with pulse pile-up correction.^80^^,^^81^ In the phantom study, cross talk was minimized by fixing ΔHbO=−ΔHb because the total concentration of hemoglobin was constant, but for *in vivo* experiments, ΔHbO and ΔHb must be fit independently. This may increase cross talk with ΔoxCCO; however, if SNR is high enough, spectral derivative analysis could be employed to minimize cross talk.^73^^,^^74^

## 5 Conclusion

This work introduced HyperTRCSS and demonstrated its potential to monitor blood oxygenation and metabolism in deep (>10  mm) tissue layers. The spectrometer can acquire a 170-point spectrum spanning 675 to 875 nm every 20 s, enabling monitoring of blood oxygenation and oxCCO over time. Homogeneous tissue-mimicking phantom experiments confirmed the sensitivity of HyperTRCSS to blood oxygenation and oxCCO changes. Further, we demonstrated in two-layer tissue-mimicking phantoms that HyperTRCSS is sensitive to changes in blood oxygenation and oxCCO beneath a 10 mm thick static top layer. Simulations suggest that the depth sensitivity of HyperTRCSS could be further enhanced if system modifications are implemented to improve the SNR.

## Data Availability

Data supporting the findings of the article are not publicly available at this time but can be obtained from the authors upon reasonable request.
