# Supplementary material for: HyperTRCSS: A hyperspectral time-resolved compressive sensing spectrometer for depth-sensitive monitoring of cytochrome-c-oxidase and blood oxygenation
Source: J Biomed Opt. 2024 Jan 24;29(1):015002. doi: 10.1117/1.JBO.29.1.015002 (PMC10807872; doi:10.1117/1.JBO.29.1.015002)
Supplement: Supplementary file 1 [file JBO_029_015002_SD001.pdf]

## Supplemental Material

*System Characterization*

Spectral calibration of the system was performed using a mercury argon lamp (HG-1, Ocean Insight, USA) and the procedure validated in Ref. 55. In brief, 13 peaks with known wavelengths (696.54, 706.72, 727.29, 738.40, 750.39, 763.51, 772.40, 800.62, 811.53, 826.45, 842.46, 851.59, and 870.87 nm) were identified in the measured spectrum. Next, the identified peaks were assigned their known wavelength values while the remaining points were determined by linear interpolation and extrapolation. The full-width half-maximum (FWHM) of the narrowest peak of the calibration lamp (763.51 nm) was considered as the spectral resolution of the system[55].

The temporal stability of the system with both lasers was characterized following a 1 h warm-up period—this is the typical time required to warm up a TR system[66,89]. DTOFs were acquired consecutively over 55 minutes, each with a collection time of 1 s. A variable neutral density filter (NDC-50C-40M, Thorlabs Inc., USA) was used to limit the photon count rate to 1% of the laser repetition rate. Figure S1 shows the temporal stability results; the YSL laser results are on the left panel (originally reported in Ref. 56) and those for the NKT laser are displayed on the right. Noticeably, the MTOF, peak intensity, FWHM, and peak position of both lasers' DTOFs were quite stable after the warm-up period.

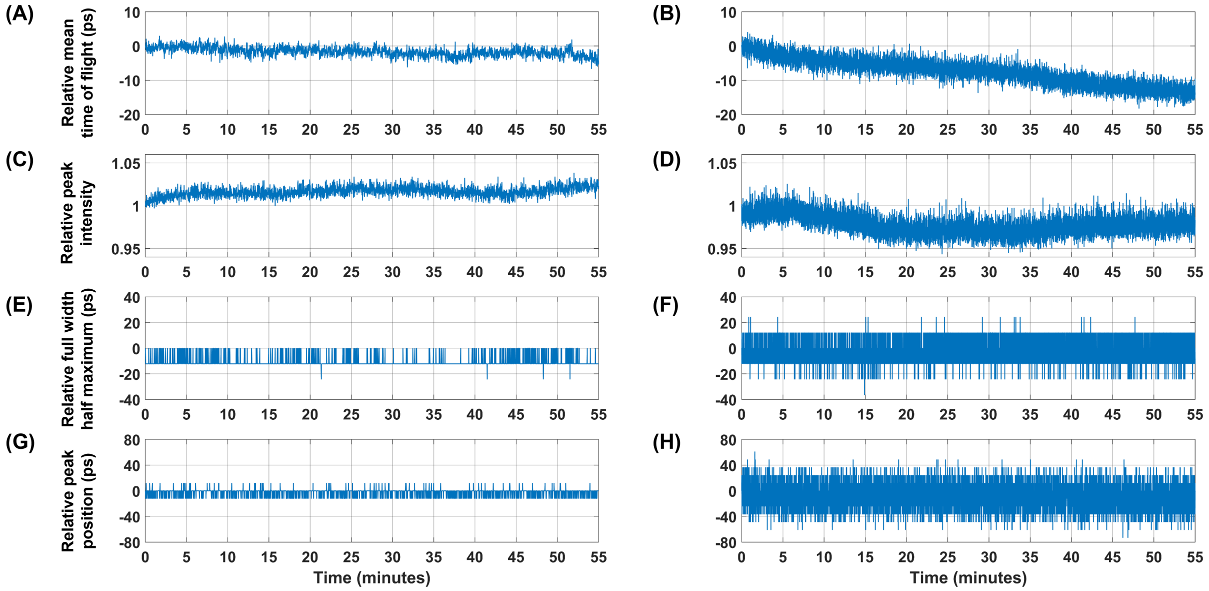


**Fig S1** Temporal stability of the YSL (left column) and NKT (right column) lasers. Relative measures are shown to allow for comparison between the lasers. (A) and (B): Mean time of flight (MTOF) in picoseconds relative to the MTOF of the first measurement in the acquisition period. (C) and (D): Peak intensity normalized by the peak intensity of the first DTOF in the acquisition period. (E) and (F): Full-width half-maximum (FWHM) in picoseconds relative to the FWHM of the first measurement in the acquisition period. (G) and (H): DTOF peak temporal position relative to the temporal position of the peak in the first DTOF in the acquisition period. The width of one time bin is $\sim$ 12 ps.

We compared the spectral stability of the lasers from startup by acquiring spectra every 30 seconds for 55 minutes using a commercial CW spectrometer (Maya2000Pro, Ocean Insight, USA). Figure S2 displays the spectral stability of the YSL laser (data from Ref. 56) and the NKT laser. The YSL laser showed remarkable spectral stability from startup while the shape of the NKT laser spectrum was only stable after 30 minutes of warm-up. Nevertheless, when the NKT laser spectral shape stabilized, it remained consistent for the remaining 20 minutes of the test.

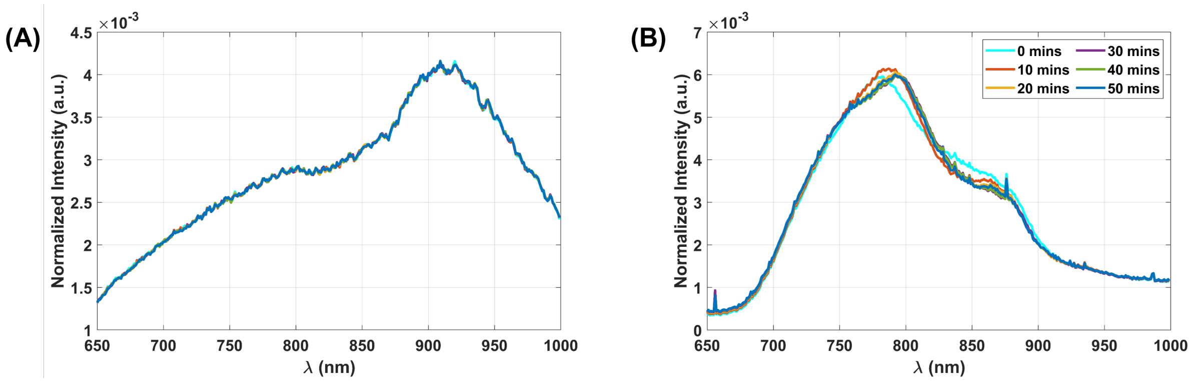


**Fig S2** Spectral stability of the YSL laser (A) and the NKT laser (B). The spectra were normalized by their area under the curve to display the spectral shape.

*Monte Carlo Simulations*

For each oxygenation level, 236 wavelengths were simulated to cover the 675–910 nm wavelength range which includes the spectral range of HyperTRCSS as well as the suggested ideal window for monitoring oxCCO.[16,22] For all simulations, the refractive index, wavelength-dependent anisotropy factor, and wavelength-dependent scattering coefficient were assigned the same values in both layers and were consistent at each oxygenation level. The refractive index was set to 1.33 in accordance with previous blood-yeast phantom literature.[60] A wavelength-dependent anisotropy factor for Intralipid-based phantoms was used.[90] The wavelength-dependent scattering coefficient was determined by fitting baseline acquisitions from an experimental phantom using the method described in Ref. 55. The scattering coefficients obtained for the baseline acquisitions were averaged and the result was fit to Eq. (S1) to recover the scattering amplitude $a$ and the scattering power $b$—800 nm was used as the reference wavelength:

$$\Delta\mu_s'(\lambda) = a\left(\frac{\lambda}{800}\right)^{-b} \tag{S1}$$

The recovered $a$ and $b$ were used in Eq. (S1) to generate the scattering coefficient at each wavelength in the simulated range. The absorption coefficients of the bottom layer at each oxygenation level were calculated according to the absolute chromophore concentrations in Table S1 and Eq. (S2):

$$\mu_a(\lambda) = Hb\varepsilon_{Hb}(\lambda) + HbO\varepsilon_{HbO}(\lambda) + H_2O\varepsilon_{H_2O}(\lambda) + oxCCO\varepsilon_{oxCCO}(\lambda) + redCCO\varepsilon_{redCCO}(\lambda) \tag{S2}$$

where Hb, HbO, $H_2O$, oxCCO, and redCCO are the concentrations of water, deoxyhemoglobin, oxyhemoglobin, oxidized cytochrome-c-oxidase, and reduced cytochrome-c-oxidase, respectively. $\mu_a(\lambda)$ is the wavelength-dependent absorption coefficient. $\varepsilon_{Hb}(\lambda)$, $\varepsilon_{HbO}(\lambda)$, $\varepsilon_{H_2O}(\lambda)$, $\varepsilon_{oxCCO}(\lambda)$, and $\varepsilon_{redCCO}(\lambda)$ are the wavelength-dependent specific extinction coefficients.[71] Note that the top

layer absorption coefficients were calculated using the concentrations in the first row of Table S1. A total hemoglobin concentration of 12 $\mu M$ was used, based on previous work with blood-yeast phantoms,[56] and the total CCO concentration was estimated based on literature values for the rat brain as we could not find estimates for yeast.[91] The saturation of hemoglobin and CCO was varied to capture the full range of chromophore concentration changes reported in Ref. 56, as we expected the experiments in this work to produce changes within that range.

**Table S1** Absolute chromophore concentrations and changes ($\Delta$) in chromophore concentration relative to simulation #1. $H_2O$ is the water fraction, Hb is the concentration of deoxyhemoglobin, HbO is the concentration of oxyhemoglobin, oxCCO is the concentration of oxidized cytochrome-c-oxidase, and redCCO is the concentration of reduced cytochrome-c-oxidase. The units of concentration for Hb, HbO, oxCCO, and redCCO are $\mu M$.

| Simulation # | $H_2O$ | Hb | HbO | oxCCO | redCCO | $\Delta Hb$ | $\Delta HbO$ | $\Delta oxCCO$ |
|---|---|---|---|---|---|---|---|---|
| 1 | 0.99 | 1 | 11 | 4.50 | 1.00 | 0 | 0 | 0 |
| 2 | 0.99 | 2 | 10 | 4.32 | 1.18 | 1 | -1 | -0.18 |
| 3 | 0.99 | 3 | 9 | 4.14 | 1.36 | 2 | -2 | -0.36 |
| 4 | 0.99 | 4 | 8 | 3.96 | 1.54 | 3 | -3 | -0.54 |
| 5 | 0.99 | 5 | 7 | 3.78 | 1.72 | 4 | -4 | -0.72 |
| 6 | 0.99 | 6 | 6 | 3.60 | 1.90 | 5 | -5 | -0.90 |
| 7 | 0.99 | 7 | 5 | 3.42 | 2.08 | 6 | -6 | -1.08 |
| 8 | 0.99 | 8 | 4 | 3.24 | 2.26 | 7 | -7 | -1.26 |
| 9 | 0.99 | 9 | 3 | 3.06 | 2.44 | 8 | -8 | -1.44 |
| 10 | 0.99 | 10 | 2 | 2.88 | 2.62 | 9 | -9 | -1.62 |
| 11 | 0.99 | 11 | 1 | 2.70 | 2.80 | 10 | -10 | -1.80 |

*Optimizing the Wavelength Range for TR Analysis*

Data from the MC simulations were used to determine the optimal wavelength range for recovering $\Delta Hb$ and $\Delta oxCCO$ from experimental TR data. We first tested the section of the "gold standard" range[16,22] captured by HyperTRCSS (780–875 nm), but found that the estimation of $\Delta Hb$ using this spectral range was poor. We then expanded the range to 680–875 nm to better capture the Hb spectral feature.[7,24] The upper bound was then adjusted from 875 nm to 870 nm, 860 nm, 850 nm, then 840 nm. Since the estimation of both chromophores became more accurate with the exclusion of the longer wavelengths, the optimal wavelength range for the TR analysis was determined to be 680-840 nm.

*Light Source Spectral Instability in Homogeneous Phantom Experiments*

It is expected that $\Delta A(\lambda)$ would return to 0 at the end of the reoxygenation period, as this represents the return of the phantom to baseline conditions. However, if there is a change in the spectral shape of the light source, $\Delta A(\lambda)$ will be non-zero despite the phantom returning to baseline conditions. This confounding effect is apparent in repetition 2 of the homogeneous phantom experiment.

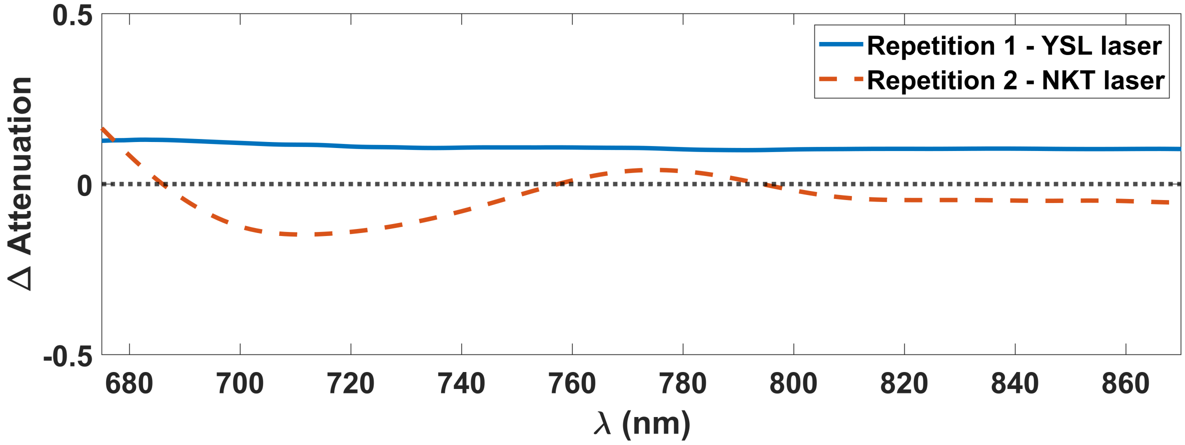


**Fig S3** Changes in attenuation $\Delta A(\lambda)$ relative to baseline (0; black dotted line) following hemoglobin reoxygenation in repetition 1 (blue solid line) and 2 (orange dashed line) of the homogeneous phantom experiment.

In repetition 2 (NKT laser), $\Delta A(\lambda)$ did not return to a flat line after reoxygenation (Fig. S3), suggesting a change in the spectral shape of the light source. In contrast, in repetition 1 (YSL laser), $\Delta A(\lambda)$ was relatively flat but non-zero at the end of reoxygenation (Fig. S3), indicating a change in the intensity of the light source but no alteration of its spectral shape.
